# Altered Pattern of Spontaneous Brain Activity in the Patients with End-Stage Renal Disease: A Resting-State Functional MRI Study with Regional Homogeneity Analysis

**DOI:** 10.1371/journal.pone.0071507

**Published:** 2013-08-22

**Authors:** Xue Liang, Jiqiu Wen, Ling Ni, Jianhui Zhong, Rongfeng Qi, Long Jiang Zhang, Guang Ming Lu

**Affiliations:** 1 Department of Medical Imaging, Jinling Hospital, Medical School of Nanjing University, Nanjing, China; 2 School of Medical Imaging, Xuzhou Medical College, Xuzhou, China; 3 Department of Nephrology, Jinling Hospital, Medical School of Nanjing University, Nanjing, China; 4 Department of Imaging Sciences, University of Rochester School of Medicine and Dentistry, Rochester, New York, United States of America; University of Maryland, College Park, United States of America

## Abstract

**Purpose:**

To investigate the pattern of spontaneous neural activity in patients with end-stage renal disease (ESRD) with and without neurocognitive dysfunction using resting-state functional magnetic resonance imaging (rs-fMRI) with a regional homogeneity (ReHo) algorithm.

**Materials and Methods:**

rs-fMRI data were acquired in 36 ESRD patients (minimal nephro-encephalopathy [MNE], n = 19, 13 male, 37±12.07 years; non-nephro-encephalopathy [non-NE], n = 17, 11 male, 38±12.13 years) and 20 healthy controls (13 male, 7 female, 36±10.27 years). Neuropsychological (number connection test type A [NCT-A], digit symbol test [DST]) and laboratory tests were performed in all patients. The Kendall's coefficient of concordance (KCC) was used to measure the regional homogeneity for each subject. The regional homogeneity maps were compared using ANOVA tests among MNE, non-NE, and healthy control groups and post hoc *t* -tests between each pair in a voxel-wise way. A multiple regression analysis was performed to evaluate the relationships between ReHo index and NCT-A, DST scores, serum creatinine and urea levels, disease and dialysis duration.

**Results:**

Compared with healthy controls, both MNE and non-NE patients showed decreased ReHo in the multiple areas of bilateral frontal, parietal and temporal lobes. Compared with the non-NE, MNE patients showed decreased ReHo in the right inferior parietal lobe (IPL), medial frontal cortex (MFC) and left precuneus (PCu). The NCT-A scores and serum urea levels of ESRD patients negatively correlated with ReHo values in the frontal and parietal lobes, while DST scores positively correlated with ReHo values in the bilateral PCC/precuneus, MFC and inferior parietal lobe (IPL) (all *P*<0.05, AlphaSim corrected). No significant correlations were found between any regional ReHo values and disease duration, dialysis duration and serum creatinine values in ESRD patients (all *P*>0.05, AlphaSim corrected).

**Conclusion:**

Diffused decreased ReHo values were found in both MNE and non-NE patients. The progressively decreased ReHo in the default mode network (DMN), frontal and parietal lobes might be trait-related in MNE. The ReHo analysis may be potentially valuable for elucidating neurocognitive abnormalities of ESRD patients and detecting the development from non-NE to MNE.

## Introduction

End stage renal disease (ESRD), an increasingly prevalent multi-symptom illness complex resulting from chronic kidney failure, has been shown to co-occur with abnormal brain function [1]. Cognitive deficits such as attention, processing speed [2], executive functions [3], motor function [4], and memory [5], [6] occur in patients with chronic kidney disease long before any overt neurological symptoms can be observed [7]. Prior studies have reported that individuals in all stages of chronic kidney disease are at higher risk for development of cognitive impairment and this may be a major determinant in their quality of life [8]–[10]. Furthermore, cognitive impairment is associated with an increased risk of death in dialysis patients [11]. Therefore, research of neuropathological mechanisms in ESRD patients may be crucial for the prompt treatment of these patients and the improvement of their prognosis.

Several studies have been carried out to investigate the structural and functional changes in ESRD. Positron emission tomography (PET) and single photon emission tomography (SPECT) are effective methods for investigating brain activity through observing changes in cerebral blood flow or cerebral metabolism [12]. Long-term hemodialysis patients without significant abnormality on neuropsychological tests showed hypo-metabolism or hypo-perfusion in the frontal cortex and thalamus [13]. Magnetic resonance spectroscopy (MRS) is ideally suited for biochemical changes in the brain and useful for monitoring of metabolic alterations [14]. The ESRD patients without clinical signs of overt encephalopathy showed metabolic disturbances in distinct brain regions as well as cognitive impairments [7]. Kim et al. used diffusion tensor imaging and found that neurologically asymptomatic patients with ESRD had abnormalities on diffusion tensor function, tractography that were associated with cognitions, including executive function attention, memory, or visuospacial function [15]. Prohovnik et al.’ study showed that ESRD patients undergoing hemodialysis have generalized cerebral atrophy and focal degeneration of the head of the caudate nucleus [16].

Recently, measures assessing resting-state brain activity with blood oxygen level dependent (BOLD) functional magnetic resonance imaging (fMRI) can reveal cognitive disorders at the early stage of the disease [17]. Thus, resting-state fMRI (rs-fMRI) has attracted more attention to investigate the spontaneous neural activity. Regional homogeneity (ReHo), as a new method, has been developed to analyze the blood oxygenation level dependent (BOLD) signal of the brain [18]. ReHo is proposed based on the hypothesis that the brain activity would more likely occur as clusters rather than as a single voxel, thus Kendall's coefficient of concordance (KCC) was used to evaluate the similarity between the time series of a given voxel and its nearest neighborhoods [19]. The pattern of resting-state brain activities obtained by the ReHo method is very similar to that observed by PET in healthy individuals, which indicates that ReHo is a promising measurement for the resting-state local brain activities [18]. ReHo has been successfully applied to study a variety of neurological and psychiatric diseases, such as epilepsy [20], social anxiety disorder [21], major depression [22], Parkinson's Disease [23], hepatic encephalopathy [24], and Alzheimer's disease [25]. To the best of our knowledge, there has been no report on the neural mechanism of cognitive function impairments in ESRD patients using rs-fMRI with ReHo algorithm. The purpose of this study was to investigate the pattern of spontaneous neural activity in ESRD patients with and without neurocognitive dysfunction using rs-fMRI with a ReHo algorithm.

## Materials and Methods

### Subjects

The study was approved by the Medical Research Ethics Committee of Jinling Hospital, Nanjing, China, and all the subjects' written informed consents were obtained before the study. The patients were recruited from patients hospitalized at Jinling Hospital, Nanjing, China. Thirty six patients (minimal nephro-encephalopathy [MNE], 13 male, 6 female, mean age 37±12.07 years; non-nephro-encephalopathy [non-NE], 11 male, 6 female, mean age 38±12.13 years) with ESRD and without overt nephro-encephalopathy were included for this study. The following exclusion criteria were as follows: (a) overt encephalopathy (episodic or persistent) as revealed by a standard clinical neurological or imaging investigation, (b) any drug/alcohol abuse history, (c) any brain lesions such as tumor, stroke assessed on basis of medical history and MRI, (d) known psychiatric disorders, (e) traumatic history, (f) head motion more than 1.0 mm or 1.0° during MR scanning. One MNE and three non-NE patients were excluded because of head motion.

Twenty healthy controls (13 male, 7 female, mean age 36±10.27 years) were recruited from the local community by advertisements. All the healthy controls had no disease of kidney or other systems, or any history of psychiatric or neurological diseases. All subjects were self-identified as right-handed with normal sight.

### Laboratory Examinations

Blood biochemistry tests, including serum creatinine and urea levels, were performed for all patients within one day before MR scanning. No laboratory tests were performed thus unavailable for the 20 normal subjects.

### Neuropsychological Tests

Neuropsychological tests, including the number connection test-A (NCT-A), digit-symbol test (DST), line-tracing test (LTT) and serial-dotting test (SDT), were performed for all subjects within 1 hours after MR scan. NCT-A examines the domain of psychomotor speed. Subjects were asked to connect figures from1 to 25 that were randomly printed on the paper as quickly as possible. A longer time to complete the test represents a worse performance. DST is associated with the domains of psychomotor speed, attention, and visual memory. Digits from 1 to 9 and corresponding symbols were displayed in front of the subjects; they were asked to fill in the blanks with the symbol that matched each figure. The more symbols correctly transcribed into the blank indicate better performance [26], [27]. Several literatures reported that neurocognitive dysfunction including the attention, processing speed [2], executive functions [3], motor function [4], and memory [5], [6] were prevalent in ESRD patients. Furthermore, NCT-A and DST scores among the three groups showed significant difference (**Table 1**). NCT-A and DST had been widely used to expose the neuropsychological impairments in hepatic encephalopathy [24], [28], [29]. Thus, MNE was defined using the NCT-A and DST neuropsychological tests. MNE was diagnosed when the scores of at least one test were beyond 2SD (standard deviation) of the mean value for the age-matched controls. According to neuropsychological tests, 19 patients were diagnosed as MNE, and 17 patients as non-NE.

**Table 1:** Demographics and clinical data of ESRD patients and healthy controls.

Protocols	MNE (n = 19)	non-NE (n = 17)	Controls (n = 20)	*P* value
Sex (M/F)	13/6	11/6	13/7	= 0.965^a^
Age (±SD), y	37±12.07	38±12.13	36±10.27	= 0.912^b^
S.creatinine (in μmol/L)	811±364.08	723±326.17		= 0.512^c^
S.urea (in mmol/L)	23.09±9.57	15.04±7.10		= 0.036^c^
Disease duration(m)	62.32±71.15	48.57±71.56		= 0.588^c^
Dialysis duration(m)	7.17±14.58	5.69±7.47		= 0.718^c^
NCT-A (s)	61.05±8.26	37.80±9.78	32.28±8.93	<0.0001^b^
DST (score)	41.47±11.70	52.40±10.38	63.22±11.54	<0.0001^b^
LTT(s)	68.69±31.41	68.43±26.30	46.56±12.15	= 0.029 b
SDT(s)	60.25±20.06	60.86±25.08	45.13±9.87	= 0.040 b

Values are expressed as mean ± SD. MNE  =  minimal nephro-encephalopathy; Non-NE  =  non-nephro-encephalopathy; NCT-A  =  number connection test-A; DST  =  digital symbol test; LTT  =  line-tracing test; SDT  =  serial-dotting test.

a The *P* value for gender distribution in the three groups was obtained by chi-square test.

b The *P* value for age and neuropsychological tests difference among the three groups were obtained by one-way analysis of variance.

c The P value for difference of serum creatinine and urea, disease and dialysis duration between the two groups was obtained by two-sample *t* test.

### MRI data acquisition

Imaging data were acquired on a 3 Tesla MR scanner (TIM Trio, Siemens Medical Solutions, Erlangen, Germany). All subjects were placed in a standard head coil and fitted to foam padding to reduce head motion. They were instructed to hold still, keep eyes closed but be awake in the MR scanner. High-resolution axial T_1_-FLASH sequence images were obtained in every subject to detect clinically silent lesions (30 axial sections; section thickness  = 4 mm; intersection gap = 0.4 mm; in-plane resolution  = 320×256; field of view (FOV)  = 240×240 mm^2^; repetition time (TR)  = 350 ms; echo time (TE)  = 2.46 ms). A gradient-echo echo-planar (GRE-EPI) sequence sensitive to BOLD contrast was used to acquire functional images (TR = 2000 ms, TE  = 30 ms, flip angle  = 90°, FOV  = 240×240 mm^2^, matrix  = 64×64, slice thickness  = 4 mm and slice gap = 0.4 mm). Each brain volume comprised 30 axial slices and each functional run contained 250 volumes. The sections were approximately along the anterior commissure–posterior commissure line and covered about −30 to 60 mm in the inferior-superior direction. Each fMRI scan lasted 500 s.

### Image Preprocessing

Image preprocessing was conducted using Statistical Parametric Mapping software (SPM8, http://www.fil.ion.ucl.ac.uk/spm/). The first ten volumes of the functional images were discarded for the signal equilibrium and participants' adaptation to the scanning circumstance. The remaining 240 time points were left for further analysis.

The slice timing, head motion correction and spatial normalization to the standard Montreal Neurological Institute (MNI) template with a resampled voxel size of 3×3×3mm^3^ were conducted. No participant had head motion of more than 1.0 mm maximum translation in any of the x, y or z directions or 1.0 degree of maximum rotation about three axes during scanning. We also evaluated the group differences in translation and rotation of head motion according to the formula 1 [30]:



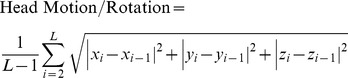
(1)where *L* is the length of the time series (*L* = 240 in this study), x*i*, y*i* and z*i* are translations/rotations at the *i*th time point in the x, y and z directions, respectively. In addition, the head motion contaminated time points were scrubbed in the same way as performed in previous studies [31]–[33]. Then, Resting State fMRI Data Analysis Toolkit (REST) Version 1.5 [34] (http://www.restfmri.net) was then used for removing the linear trend of time courses and for temporally band-pass filtering (0.01–0.08 Hz) [35] to reduce low-frequency drift and physiological high frequency respiratory and cardiac noise.

### ReHo analysis

The ReHo analysis was performed for each subject by the Resting State fMRI Data Analysis Toolkit (REST) software [34]. A Kendall's coefficient of concordance (KCC) value (also called ReHo value) was calculated to measure the similarity of the ranked time series of a given voxel to its nearest 26 neighbor voxels in a voxel-wise way with the formula 2:
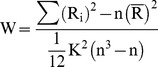
(2)where W is the KCC for a given voxel, ranging from 0 to 1; R*i* is the sum rank of the *i*th time point; 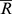 =  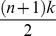
is the mean of the R*i*s; k is the number of time series within a measured cluster (27, one given voxel plus the number of its neighbors); n is the number of ranks (here, n = 240 time points).

Through calculating the KCC value of every voxel in the whole brain, an individual ReHo map was obtained for each subject. The intracranial voxels were extracted to make a mask [36]. For standardization purposes, each individual ReHo map was divided by its own mean ReHo within the mask. Spatial smoothing was then performed with an 8-mm full-width at half-maximum (FWHM) Gaussian kernel.

### Statistics analysis

Statistical analysis was performed using the software SPSS version 16.0 (SPSS Inc. Chicago, IL) for demographic and clinical data, and SPM8 (statistical parametric mapping, http://www.fil.ion.ucl.ac.uk/spm/) for fMRI data. A second-level random-effect one-sample *t* test was performed to show the ReHo results for each group (MNE, non-NE and healthy control), the threshold was set at *P*<0.01, corrected with false discovery rate (FDR) criterion. To explore the ReHo differences among the three groups (MNE, non-NE patients, and healthy controls), a one-way analysis of variance (ANOVA) was performed on the individual normalized ReHo maps in a voxel-by-voxel manner. Age and gender were included as covariates in the present and following functional data statistic analysis. The result was corrected using the Alphasim program, which setting at *P*<0.01 and cluster size >189 mm^3^, which corresponded to a corrected *P*<0.05. If statistical difference was present, post hoc *t*-tests were performed to detect the inter-group difference of brain regions. A multiple regression analysis was used to investigate the relationship between ReHo values in ESRD patients and the NCT-A/DST scores, serum creatinine and urea levels, disease duration and dialysis duration in SPM8. The threshold was set at *P*<0.05 (AlphaSim corrected).

## Results

Demographics and clinical data for ESRD patients and healthy subjects were summarized in Table 1. No significant differences were found for age (*P* = 0.912) and gender (*P* = 0.965) among the three groups. Serum creatinine values between the two patient groups didn't reach the statistical significance (*P* = 0.512). There were no significant differences for SDT (*P* = 0.951) and LTT (*P* = 0.981) scores between two patient groups, although two patient groups got higher scores of SDT and LTT than healthy controls. However, serum urea level in MNE group was significantly higher than non-NE group's (*P* = 0.036). NCT-A and DST scores among the three groups showed significant difference (both *P*<0.01). The MNE patients (61.05±8.26 sec) took longer time than non-NE patients (37.80±9.78 sec) in performing NCT-A (*P*<0.001), while MNE group (41.47±11.70 scores) had lower DST scores than non-NE group (52.4±10.38 scores) (*P* = 0.007).

**Figure 1:**
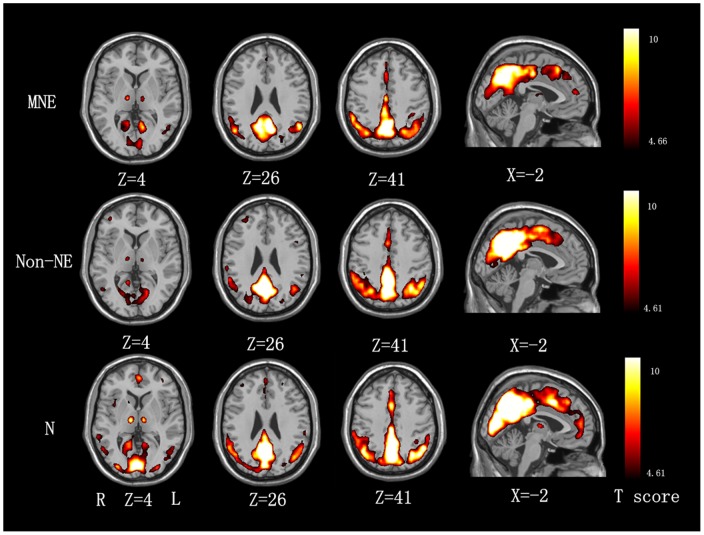
Mean ReHo maps within the MNE, non-NE and healthy control groups (P<0.01, FDR corrected). The PCC/precuneus, MPFC, IPL, bilateral middle/inferior temporal gyrus, cuneus and thalamus exhibit significantly higher ReHo values than the global mean ReHo value within each group, and with a gradually decreasing tendency from normal controls, non-NE to MNE. MNE  =  minimal nephro-encephalopathy; non-NE  =  non-nephro-encephalopathy; ReHo  =  regional homogeneity; FDR  =  false discovery rate; PCC  =  posterior cingulate cortex; MPFC  =  medial prefrontal cortex; IPL  =  inferior parietal lobe.

ReHo results within each group are shown in Fig. 1 (*P*<0.01, FDR correction). The default mode network (DMN) including the precuneus/posterior cingulate cortex (PCC), medial prefrontal cortex (MPFC), inferior parietal lobe (IPL), bilateral middle/inferior temporal gyrus and other regions including the cuneus and thalamus exhibited significantly higher ReHo values than the global mean ReHo value, and with a gradually decreasing tendency from normal controls, non-NE to MNE. (**Fig.** **1**).

**Figure 2:**
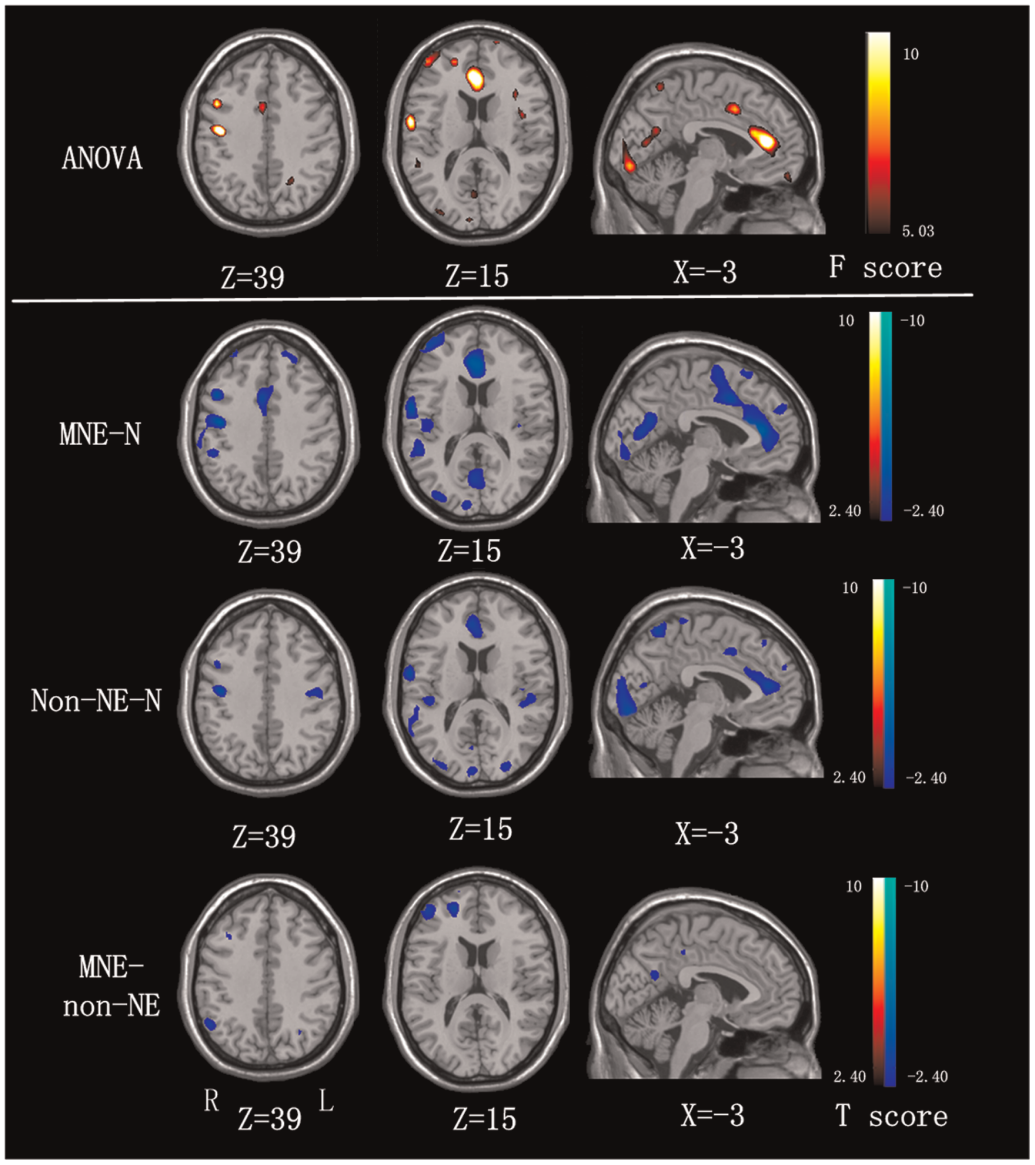
ReHo differences among MNE, non-NE, and healthy controls (P<0.05, AlphaSim corrected). Compared with the healthy controls, MNE patients show decreased ReHo in the bilateral SMA, ACC, PCC, MOG, right insula, cuneus, MFG, IPL, STG, PCu, and left PreCG, PoCG, and non-NE patients show decreased ReHo in the bilateral ACC, cuneus, precuneus, STG, PreCG, left PoCG, MOG and right MFG, and Compared with the non-NE patients, the MNE patients show decreased ReHo in the right IPL, MFG and left precuneus. MNE  =  minimal nephro-encephalopathy; non-NE  =  non-nephro-encephalopathy; ReHo  =  regional homogeneity; IPL  =  inferior parietal lobe; PCu  =  precuneus; ACC  =  anterior cingulate cortex; PCC  =  posterior cingulate cortex; SMA  =  supplementary motor area; PreCG  =  precentral gyrus; PoCG  =  postcentral gyrus; SFG  =  superior frontal gyrus; MFG  =  medial frontal gyrus; STG  =  superior temporal gyrus; MOG  =  medial occipital gyrus.

Compared with the healthy control group, both MNE and non-NE patients showed significantly decreased regional homogeneity in the bilateral frontal, parietal and temporal lobes (**Fig.** **2** **,** **Tables** **2** **and** **3**). In addition, when comparing with the non-NE patients, MNE patients showed decreased regional homogeneity in the right inferior parietal lobe (IPL), medial frontal cortex (MFC) and left precuneus (PCu). (*P*<0.05, AlphaSim corrected) (**Fig.** **2** **,** **Table** **4**).

**Table 2:** Regions showing ReHo differences between MNE patients and healthy controls.

Brain regions	BA	MNI coordinates (mm)	Vol? (mm^3^)	Peak t value
		(x, y, z)		
SMA(R+L)	32	3,21,30	3483	−2.40
ACC(R+L)	32	−3,48,0	6993	−2.40
PCC(R+L)	23	0, −27,33	972	−2.52
cuneus(R)	7/19	6, −78, −24	1917	−2.41
MFG(R)	24	48. −27,48	4644	−2.40
IPL(R)	4/6	42, −27,15	3618	−2.40
PCu(R)	7	41, −48,43	5265	−2.40
STG(R)	14	45, −9,59	486	−2.40
Insula(R)	13	45, −9,45	999	−2.40
MOG(R)	19	30, −87,9	1242	−2.42
SFG(L)	10/46	−21,51,42	729	−2.42
MOG(L)	19	−36, −75,33	1242	−2.43
PreCG(L)	4/6	−54, −6,48	702	−2.43
PoCG(L)	40/3	−48, −18,51	891	−2.43

Negative sign represents decrease. ReHo  =  regional homogeneity; MNI  =  Montreal Neurological Institute; SMA  =  supplementary motor area; ACC  =  anterior cingulate cortex; PCC  =  posterior cingulate cortex; SFG  =  superior frontal gyrus; PCu  =  precuneus; MFG  =  medial frontal gyrus; IPL  =  inferior parietal lobule; PoCG  =  postcentral gyrus; PreCG  =  precentral gyrus; STG  =  superior temporal gyrus; MOG  =  middle occipital gyrus; BA  =  brodmann area. P<0.05, Alphasim corrected.

**Table 3:** Regions showing ReHo differences between non-NE patients and healthy controls.

Brain regions	BA	MNI coordinates (mm)	Vol? (mm^3^)	Peak t value
		(x, y, z)		
MFG(R)	24	42,15,33	2295	−2.41
ACC(R+L)	32	6,30,24	2943	−2.42
PreCG(R)	4/6	60, −9,18	1323	−2.41
PreCG(L)	4/6	−59, −18,36	1755	−2.41
cuneus(R+L)	7/19	0, −75, −18	3483	−2.40
PoCG(L)	40/3	−45, −39,51	1269	−2.43
PCu(R)	7	9, −63,60	756	−2.41
PCu(L)	7	−9, −51,66	2106	−2.41
STG(R)	14	39, −24,9	2133	−2.41
STG(L)	14	−54, −24,15	1539	−2.41
MOG(L)	19	−30, −93,18	2214	−2.43

Negative sign represents decrease. ReHo  =  regional homogeneity; MNI  =  Montreal Neurological Institute; MFG  =  medial frontal gyrus; ACC  =  anterior cingulate cortex; PCu  =  precuneus; PreCG  =  precentral gyrus; PoCG  =  postcentral gyrus; STG  =  superior temporal gyrus; MOG  =  middle occipital gyrus; BA  =  brodmann area. P<0.05, Alphasim corrected.

**Table 4:** Regions showing ReHo differences between MNE and non-NE patients.

Brain regions	BA	MNI coordinates (mm)	Vol? (mm^3^)	Peak t value
		(x, y, z)		
MFG(R)	24	48,51,9	2403	−2.40
IPL(R)	4/6	48, −54,39	945	−2.43
PCu(L)	7	−12, −63,18	459	−2.43

Negative sign represents decrease. ReHo  =  regional homogeneity; MNI  =  Montreal Neurological Institute; MFG  =  medial frontal gyrus; IPL  =  inferior parietal lobule; PCu  =  precuneus; BA  =  brodmann area. P<0.05, Alphasim corrected.

**Figure 3:**
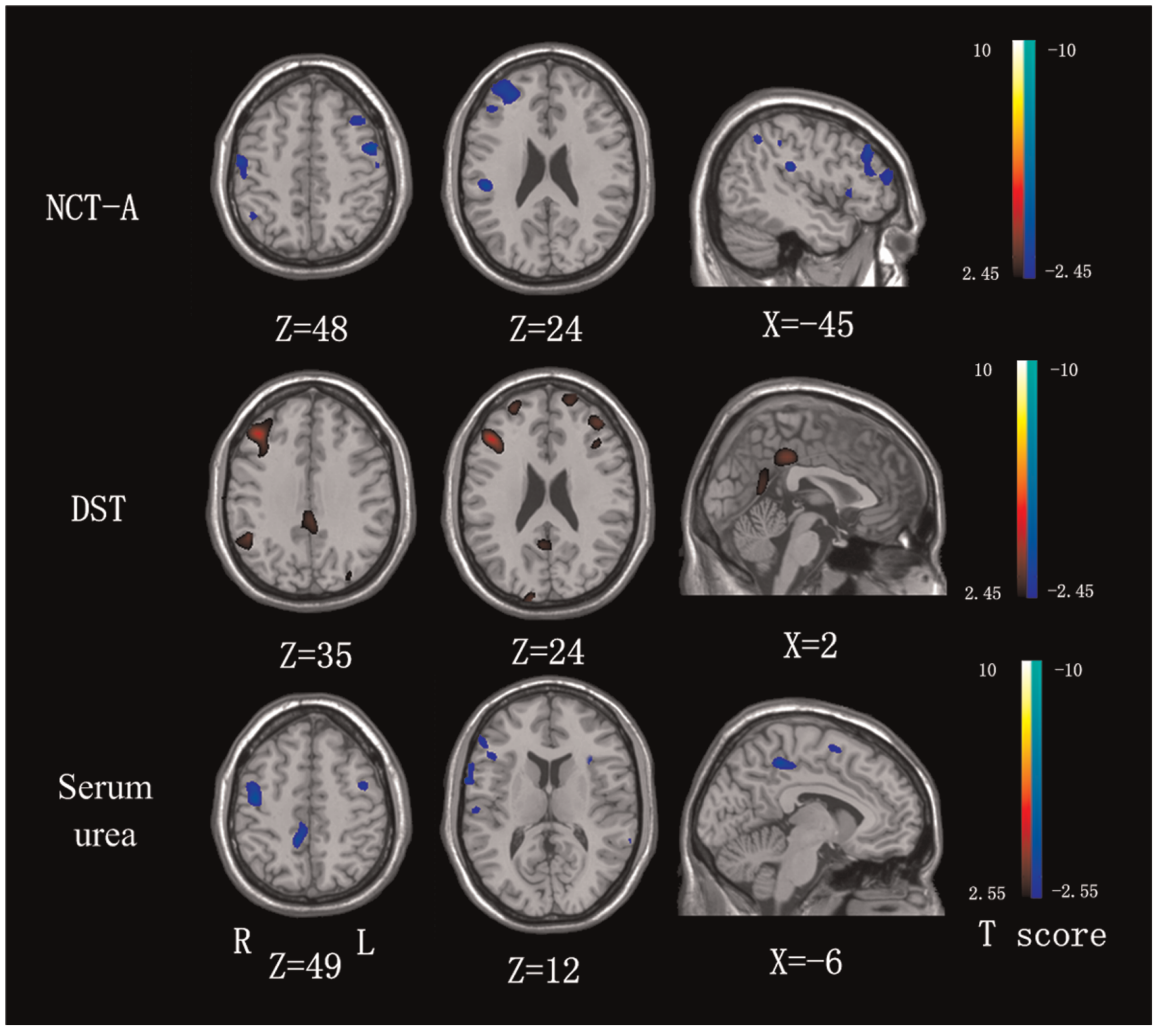
The correlation between the ReHo values and NCT-A/DST scores, as well as serum urea levels in the combined MNE and non-NE groups (P<0.05, AlphaSim corrected). The NCT-A has a negative correlation with the ReHo values in the bilateral frontal and parietal lobes, including medial frontal cortex (MFC), superior frontal cortex (SFC), precentral gyrus and postcentral gyrus. The DST scores positively correlate with ReHo values in the bilateral PCC/precuneus, medial frontal cortex (MFC) and inferior parietal lobe (IPL). Serum urea levels negatively correlated with the ReHo values in the right inferior frontal gyrus (IFG), precuneus, MFC, precentral gyrus and postcentral gyrus. NCT-A  =  number connection test type A; DST  =  digital symbol test; ReHo  =  regional homogeneity.

Correlation analysis of ReHo at each voxel in the whole brain against the NCT-A results of ESRD patients revealed negative correlation in the bilateral frontal and parietal lobes, including medial frontal cortex (MFC), superior frontal cortex (SFC), precentral gyrus and postcentral gyrus **(** **Fig.** **3**). DST scores positively correlated with ReHo values in the bilateral PCC/precuneus, medial frontal cortex (MFC) and inferior parietal lobe (IPL) (*P*<0.05, AlphaSim corrected) (**Fig.** **3**). Serum urea of ESRD patients negatively correlated with ReHo values in the right inferior frontal gyrus (IFG), precuneus, MFC, precentral gyrus and postcentral gyrus (*P*<0.05, AlphaSim corrected) (**Fig. 3**). No significant correlations were found between any regional ReHo values and disease duration, dialysis duration and serum creatinine values in ESRD patients (all *P*>0.05, AlphaSim corrected).

## Discussion

In this study, we found diffusely decreased ReHo values in cortical regions in both MNE and non-NE patients compared with healthy control group, which correlated with neuropsychological impairments in these ESRD patients. Importantly, patients with MNE showed more widespreadly decreased ReHo values in some brain areas than non-NE patients, suggesting further impairment with the development from non-NE to MNE.

An important finding in this study is that compared with non-NE, the MNE patients show decreased ReHo values mainly in the right inferior parietal lobe (IPL), medial frontal cortex (MFC) and left precuneus (PCu), all of which are important components of default-mode network (DMN) [37], [38]. It has been reported that the DMN regions were recruited in a wide spectrum of visual and auditory attention, language processing, memory, and motoric activity [39]. In addition, the neuropsychological tests used in our study including NCT-A and DST [26], [27], which were associated with the domains of psychomotor speed and domains of psychomotor speed, attention, and visual memory, were correlated with ReHo values in the special brain regions of DMN. These neuro-cognitive domains are consistent with the clinical studies about substantial impairment in patients with ESRD [2], [4]–[6]. Taken together, our study suggests the role of DMN areas in the development from non-NE to MNE in the ESRD patients.

We also found decreased ReHo in the frontal and parietal cortex, which was consistent with previous studies [1], [13]. Parietal cortex lesions are considered to be engaged in distinct attention functions such as attention shift, visuo-spatial attention, working memory, and supramodal control [40]–[42]. Frontal lobe dysfunction is characterized clinically by decreased spontaneity, initiative, insight, judgment, abstraction, perseverance and response inhibition [43]. These abnormal behavioral features are common in patients with chronic renal failure. There are several possible explanations why ESRD patients may preferentially develop frontal-subcortical cognitive deficits. First, selective cognitive problems in patients may result from ESRD comorbidities (e.g., diabetes, hypertension) as well as their treatment, most of them shown to predominantly impact fronto-subcortical systems [44]; Second, white matter hyperintensities have been shown to preferentially impair frontal lobe function regardless of their location [45]. Third, many ESRD patients undergo dialysis, which are still not able to remove all the toxins, middle-large molecules in particular [46], and, for unknown reasons, these toxins have been shown to particularly affect frontal white matter [47]. We also found serum urea of ESRD patients negatively correlated with ReHo values in the frontal and parietal lobes including inferior frontal gyrus (IFG), MFC, precuneus, precentral gyrus and postcentral gyrus (*P*<0.05, AlphaSim corrected). The serum urea level may play an important role in development of MNE, especially in the dysfunction of the frontal and parietal lobes.

We acknowledged our study had some limitations. First, this study is preliminary and our results are limited to a small sample size with heterogeneous patient etiology, which may affect the statistical analysis and comprehensive interpretation of the results. Further studies with large-cohort and homogeneous etiology are needed. Second, some cognitive impairments in ESRD patients are related to ESRD itself and others are secondary to dialysis [48], therefore the dialysis is possibly a crucial factor that lead to cognitive impairment, further studies about whether dialysis or not and the pattern of dialysis (hemodialysis or peritoneal dialysis) should be considered. Third, as a cross-sectional study, we can only observe the progression from non-NE to MNE in different subjects but not in the same patient group recruited in a longitudinal study. A truly longitudinal study is warranted to confirm the finding of this study. Forth, although some neuropsychological tests used here to define the MNE may not be special for the ESRD patients, our findings indicated NCT-A and DST can be used to evaluate neurocognitive dysfunction in ESRD patients.

In conclusion, the present study showed that the ReHo method could detect patterns of homogeneity changes in patients with ESRD in the resting state. Diffused decreased ReHo values were found in both MNE and non-NE patients, which correlated with neuropsychological impairments in these ESRD patients. The progressively decreased ReHo in the DMN, frontal and parietal lobes might be trait-related in MNE. The ReHo analysis may be potentially valuable to explore the pathophysiology of the ESRD and detect the development from non-NE to MNE.
